# RNA-Sequencing Reveals the Involvement of Sesquiterpene Biosynthesis Genes and Transcription Factors during an Early Response to Mechanical Wounding of *Aquilaria sinensis*

**DOI:** 10.3390/genes14020464

**Published:** 2023-02-11

**Authors:** Jieru Xu, Ruyue Du, Yue Wang, Jinhui Chen

**Affiliations:** 1 Sanya Nanfan Research Institute of Hainan University, Hainan Yazhou Bay Seed Laboratory/School of Forestry, Hainan University, Sanya 572019, China; 2 Key Laboratory of Genetics and Germplasm Innovation of Tropical Special Forest Trees and Ornamental Plants, Ministry of Education/Engineering Research Center of Rare and Precious Tree Species in Hainan Province, School of Forestry, Hainan University, Haikou 570228, China

**Keywords:** *Aquilaria sinensis*, RNA-seq, sesquiterpenoid biosynthesis, mechanical wounding response, agarwood formation

## Abstract

Plants respond to wounding by reprogramming the expression of genes involved in secondary metabolism. *Aquilaria* trees produce many bioactive secondary metabolites in response to wounding, but the regulatory mechanism of agarwood formation in the early response to mechanical wounding has remained unclear. To gain insights into the process of transcriptome changes and to determine the regulatory networks of *Aquilaria sinensis* to an early response (15 days) to mechanical wounding, we collected *A. sinensis* samples from the untreated (Asc1) and treated (Asf1) xylem tissues and performed RNA sequencing (RNA-seq). This generated 49,102,523 (Asc1) and 45,180,981 (Asf1) clean reads, which corresponded to 18,927 (Asc1) and 19,258 (Asf1) genes, respectively. A total of 1596 differentially expressed genes (DEGs) were detected in Asf1 vs. Asc1 (|log_2_ (fold change)| ≥ 1, P_adj_ ≤ 0.05), of which 1088 were up-regulated and 508 genes were down-regulated. GO and KEGG enrichment analysis of DEGs showed that flavonoid biosynthesis, phenylpropanoid biosynthesis, and sesquiterpenoid and triterpenoid biosynthesis pathways might play important roles in wound-induced agarwood formation. Based on the transcription factor (TF)-gene regulatory network analysis, we inferred that the bHLH TF family could regulate all DEGs encoding for farnesyl diphosphate synthase, sesquiterpene synthase, and 1-deoxy-D-xylulose-5-phosphate synthase (DXS), which contribute to the biosynthesis and accumulation of agarwood sesquiterpenes. This study provides insight into the molecular mechanism regulating agarwood formation in *A. sinensis*, and will be helpful in selecting candidate genes for improving the yield and quality of agarwood.

## 1. Introduction

Plants produce various secondary metabolites that act as tools for them to deal with adverse environments and survive well [1]. Meanwhile, many metabolites are used for their pharmaceutical properties, as food additives, and in aromatic and culinary purposes. Owing to the huge economic value of secondary metabolites, increasing attention has been paid to maximizing their yield with the help of modern molecular techniques [2]. However, information on how plants produce secondary metabolites in response to environmental stimuli, including agarwood formation, is still limited.

Agarwood is a highly valuable aromatic resin, which is produced by *Aquilaria* trees as a non-specific host response to mechanical wounding, insect attack, or microbial invasion [3]. Agarwood is extensively used in precious drugs, religious practices, and in cultural activities. Agarwood is also popular as a precious perfume and an incense in many Asian countries, such as India, Japan, and China. More than 300 chemical compounds in agarwood have been characterized, including sesquiterpenes, and 2-(2-phenylethyl)chromones, as well as flavonoids [4]. In agarwood extracts, sesquiterpenes and 2-(2-phenylethyl)chromones, as the predominant components, show various pharmacological activities, such as antibacterial, antifungal, and anti-inflammatory properties [4]. Additionally, the content and concentration of sesquiterpenes can be the criteria to evaluate the quality of agarwood [4,5]. Increasing attention has been paid recently to methods that induce agarwood formation [6,7,8,9]. However, the quality and yield of agarwood after treatment with artificial methods, including the burn-chisel-drill method [6], formic acid treatment [8], and fungus infestation [3], are not satisfactory. In addition, high-quality agarwood develops slowly over time, even several hundred years, and yet has growing market demand throughout the world, making it the wood of gods. Therefore, a better understanding of the agarwood formation at the molecular level would be helpful in improving the quality and yield of agarwood.

Agarwood formation is closely associated with programmed cell death [10,11]. During this process, the activity of the ray parenchyma cells would be triggered, leading to the consumption of reserve materials [12,13]. The living parenchyma cells convert starch grains into sesquiterpenes, chromone derivatives, phenolic substances, and other components [13]. As a result, these substances are mainly detected in the agarwood layer and transition layer, followed by the agarwood-normal transition layer [13]. Similar results were also reported for the heartwood formation in other species, such as *Santalum album* [14], *Pinus sylvestris* [15,16], and *Taiwania cryptomerioides* [17]. These studies provide useful information for further study on the molecular mechanism underlying agarwood formation.

Many studies concentrated on the functional identification and regulation of key genes that could contribute to the agarwood formation in response to physical, chemical, and biological induction, including genes encoding transcription factors (TFs), sesquiterpene and 2-(2-phenylethyl)chromone biosynthetic enzymes, as well as other potential factors. For example, a recent study indicated that *Aquilaria sinensis* calli, under salinity stress, could produce 2-(2-phenylethyl)chromones, as the salt stress induced dynamic changes in transcriptional levels of genes encoding chalcone synthases and O-methyltransferases that triggered their biosynthesis [18]. Transcriptome analysis of healthy and wounded tissues of *A*. *sinensis* demonstrated that 30 genes, potentially encoding enzymes in the sesquiterpene biosynthesis pathway, were associated with agarwood formation [19]. These included 3-hydroxy-3-methylglutaryl-coenzyme A reductase (HMGR), farnesyl diphosphate (FPP) synthase (FPPS), sesquiterpene synthase (SS), and 1-deoxy-D-xylulose-5-phosphate synthase (DXS) [19]. Additionally, several TFs also regulate the key genes involved in this process of agarwood formation [20,21,22]. AsMYC2, a basic helix-loop-helix TF, activated *ASS1* expression through the jasmonic acid signaling pathway during the biosynthesis of agarwood sesquiterpenes in wounded *Aquilaria sinensis* [23]. The ERF TF, AsERF1, participated in the sesquiterpene biosynthesis by interacting with the promoter to increase the *AsTPS1* expression level [21]. Despite these discoveries, the molecular mechanism of agarwood formation, as an early response to wounding, has yet not been well-elucidated.

The formation of agarwood is closely associated with the response of *Aquilaria* trees to wounding, and this process takes a long time. A previous study on the agarwood process investigated different wood samples from the three post-wound stages, including the early (hours to 14 days), middle (4–24 weeks), and late (7–12 months) stages, and the result showed that these woods could turn from light yellow to brown to dark with the treatment time [24]. Total phenols and terpenes increased significantly over time [25]. Hence, we select the time point (15 days) in the early stage of the agarwood process to investigate the early response mechanism to wounding. We aim to analyze the responsive genes of specific xylem tissues of *A. sinensis* (Lour.) Gilg after mechanical wounding by carrying out RNA-sequencing (RNA-seq), gaining some insights into the molecular mechanism of agarwood formation. Our study identifies the co-expression network and TFs that potentially participate in a regulatory network controlling sesquiterpene biosynthesis.

## 2. Materials and Methods

### 2.1. Plant Material and RNA Extraction

Five-year-old *A. sinensis* trees, grown in an artificial nursery in Hainan Province (19°38′56″ N, 110°14′29″ E), were used. The stems of three such trees at a height of 1 m were wounded by a chisel to induce agarwood formation, leading to the fan-shaped wound with about 4 cm depth. Fifteen days after the mechanical wounding (early response), the treated and untreated xylem tissues (1 m above the wounded site) were collected per tree for three such trees, and defined as Asf1 (the treatment) and Asc1 (the control), respectively. A total of six samples (Asf11, Asf12, Asf13; Asc11, Asc12, Asc13) were immediately frozen in liquid nitrogen and stored at −80 °C until RNA extraction. Total RNA was extracted with the help of an RNAprep pure plant plus kit (Tiangen, Beijing, China). RNA quality was monitored on a NanoDrop 6000 Assay Kit of the Bioanalyzer 2100 system (Agilent Technologies, CA, USA).

### 2.2. Transcriptome Profiling of the Wounded and Healthy Xylem Tissues from A. sinensis

The isolation of poly(A)-enriched mRNA from total RNA was obtained with the help of a fragmentation buffer to produce shorter strands. Random hexamer primers as well as M-MuLV reverse transcriptase were used for the synthesis of first-strand cDNA, followed by second-strand cDNA synthesis by DNA polymerase I, and dNTPs. After adenylation of 3′ ends and ligation adaptors of DNA fragments, the library fragments were purified with the help of an AMPure XP system (Beckman Coulter, Beverly, CA, USA) to choose cDNA fragments ranging from 370 to 420 bp. Finally, the six cDNA libraries were PCR-enriched and sequenced by the Illumina HiSeq 6000 System.

The percentage of nucleotides with a quality value of more than 20 (Q20) and 30 (Q30), and the GC content of the clean data, were calculated. Clean reads of the six libraries were aligned to the *A. sinensis* reference genome [26] by using Hisat2 (v 2.0.5) [27]. The read number of genes was calculated using featureCounts v1.5.0-p3 [28]. Gene expression levels were represented by fragments per kilobase of transcript per million fragments mapped reads (FPKM). Differential expression analysis was performed with the help of the DESeq2 R package (1.20.0) [29]. The Benjamini and Hochberg’s approach was applied to correct *p*-values to supervise the false discovery rate [30]. Genes with an adjusted *p*-value (P_adj_) ≤ 0.05 and |log_2_ (fold change)| ≥ 1 were identified as significantly differentially expressed between Asf1 and Asc1. All the differentially expressed genes (DEGs) were mapped to the Gene Ontology (GO) database (http://www.geneontology.org/) and the Kyoto Encyclopedia of Genes and Genomes (KEGG) database (http://www.genome.jp/kegg/). The gene functional analysis was performed on the clusterProfiler R package (3.8.1) [31]. A P_adj_ ≤ 0.05 was considered as significant.

### 2.3. Correlation Networks

Based on the FPKM values of the DEGs involved in sesquiterpene biosynthesis and the TF genes, co-expression analysis between genes and TFs was examined using Pearson’s correlation coefficient (cor), calculated in R studio. The TF-gene pairs (|cor| ≥ 0.9 and *p*-value < 0.05) were considered as significant co-expression, displaying the transcriptional regulatory network using Cytoscape (v 3.7.2) [32]. The *p*-value of the correlation was calculated by the permutation test method [33].

### 2.4. qRT-PCR Analysis

Validation of eight selected genes with quantitative real-time polymerase chain reaction (qRT-PCR) was used to confirm the RNA-seq results. The gene primers (Table S1) were designed by the Primer Premier 5.0 software. cDNA was synthesized from total RNA with the help of TB Green^®^ Premix Ex Taq™ (Tli RNaseH Plus; Takara, Beijing, China). The qRT-PCR analysis was performed in a 20 µL volume: 10 µL of 2 × SYBR Premix Ex Taq, 0.8 µL of each primer, 0.4 µL of ROX Reference II, 2 µL of the cDNA temple, and 6 µL of ddH_2_O. The reaction conditions were 94 °C for 2 min; 40 cycles of 95 °C for 5 s; and 60 °C for 30 s. The *Ubiquitin* of *A. sinensis* was selected as the endogenous control gene. The calculation of relative expression levels of genes was conducted using the 2^-∆∆CT^ method [34]. The log_2_(fold change) of qRT-PCR was analyzed and compared with that of RNA-seq. The fold change describes the ratio of two values, Asf1/Asc1.

## 3. Results

### 3.1. Global Analysis of Transcriptome of A. sinensis

**Figure 1:**
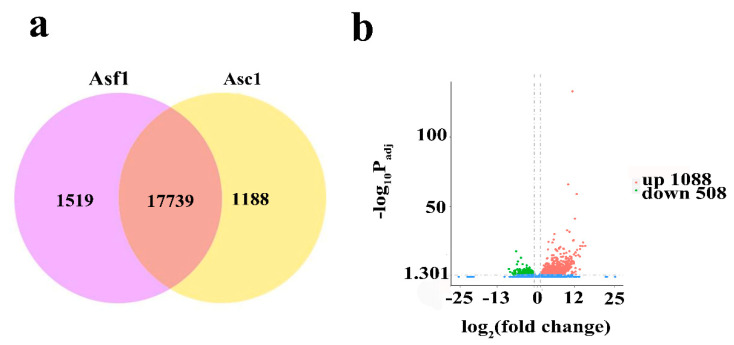
Global analysis of gene expression in specific xylem tissues of wound-induced agarwood formation. (**a**) The Venn diagram shows common and unique expressed genes in Asf1 and Asc1; (**b**) the number of up-regulated and down-regulated DEGs from Asf1 vs. Asc1. We judged the significance of gene expression difference with P_adj_ ≤ 0.05 and |log_2_ (fold change)| ≥ 1.

In a full-scale sequencing analysis of six cDNA libraries (Table 1), we obtained an average of 49,102,523 (Asc1), and 45,180,981 (Asf1) clean reads. Overall, the mapping ratios of Asc1 and Asf1 were 91.38%, and 87.71%, respectively (Table 1), which corresponded to 18,927 (Asc1) and 19,258 (Asf1) genes, respectively (Figure 1a). Among these, 1188 and 1519 genes were unique to Asc1 and Asf1, respectively, and 17,739 genes were expressed in Asf1 and Asc1 (Figure 1a). Additionally, the Q20 and Q30 were 98.04% (Asc1) and 98.05% (Asf1), 94.13% (Asc1) and 94.24% (Asf1), respectively (Table 1), whereas the GC contents were 46.59% (Asc1) and 46.84% (Asf1) (Table 1). These results indicated that clean data were of high quality for subsequent quantitative analysis. A total of 1596 DEGs were detected in Asf1 vs. Asc1, of which 1088 were up-regulated and 508 DEGs were down-regulated (Figure 1b). The data show that the agarwood formation, after mechanical wounding, involves a large-scale reprogramming of transcriptome, which indicates the involvement of several biological events.

### 3.2. Functional Enrichment Analysis of DEGs

**Figure 2:**
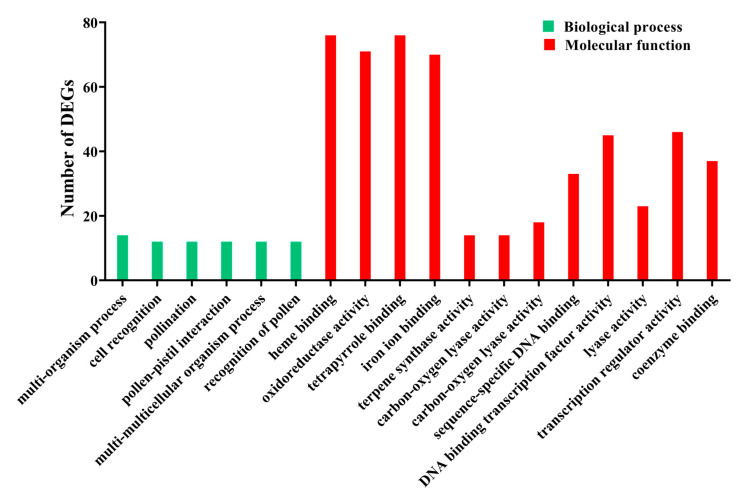
The annotation of DEGs in GO terms. The horizontal axis shows the two GO categories: the biological process, and molecular function; the vertical axis shows the number of DEGs annotated under each term.

GO enrichment analysis was performed to identify the biological functions of 1596 DEGs. A total of 18 GO terms were significant (P_adj_ ≤ 0.05). For the ‘biological process’ category, six terms related to ‘multi-organism process’, ‘cell recognition’, ‘pollination’, ‘pollen-pistil interaction’, ‘multi-multicellular organism process’, and ‘recognition of pollen’ were obtained (Figure 2). Within the ‘molecular function’ category, groups related to ‘heme binding’, ‘tetrapyrrole binding’, and ‘oxidoreductase activity’ as well as ‘iron ion binding’ were identified (Figure 2). No significant GO terms were identified in the category of ‘cellular component’ for DEGs. These results indicate that various biochemical processes may be triggered in *A*. *sinensis* as an early response to wounding. To better understand their function, 1596 DEGs were annotated into the KEGG database. In particular, 80 DEGs were significantly mapped to seven pathways with P_adj_ ≤ 0.05, including 14 in the flavonoid biosynthesis pathway; 24 in the phenylpropanoid biosynthesis pathway; 12 in the glutathione metabolism pathway; 6 in the sesquiterpenoid and triterpenoid biosynthesis pathway; 8 in the phenylalanine, tyrosine, and tryptophan biosynthesis pathway; 7 in the phenylalanine metabolism pathway; and 9 in the photosynthesis pathway (Table 2).

### 3.3. DEGs Involved in Hormone Signal Transduction

**Figure 3:**
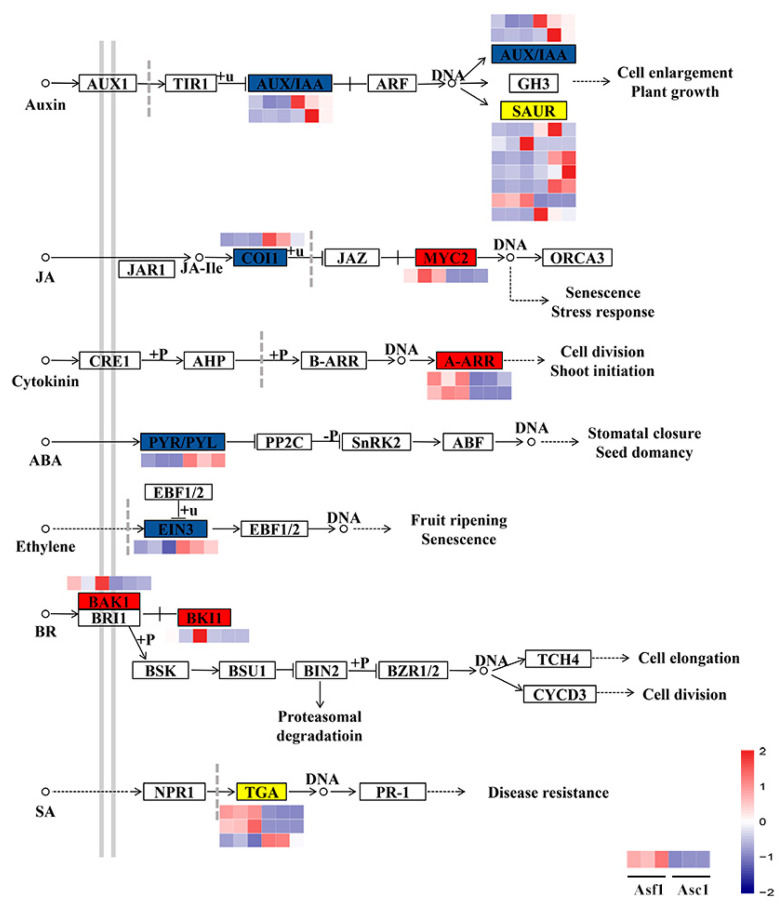
Expression profiling of the DEGs in the hormone signal transduction pathway. The boxes of up-regulated DEGs are marked in red, the down-regulated DEGs are marked in blue, and both are marked in yellow. The expression levels of the related DEGs are shown in the heatmap beside the boxes. Each row represents each DEG, and each column represents the expression level from different samples. The color from blue to red represents gene expression from low to high.

A total of 20 DEGs associated with hormone signal transduction were detected, which included auxin, jasmonic acid (JA), cytokinin, abscisic acid (ABA), ethylene, brassinosteroid (BR), and salicylic acid (SA) (Figure 3; Table S2). In the auxin signal transduction pathway, two DEGs that encoded for AUX/IAA were down-regulated (Figure 3; Table S2). Of the seven SAUR-encoding DEGs, only two were up-regulated, whereas five were down-regulated (Figure 3; Table S2). In the JA signal transduction pathway, a *coronatine insensitive 1* (*COI1*) was down-regulated, while the *MYC2* gene was up-regulated (Figure 3; Table S2). In the cytokinin signal transduction pathway, two *type-A response regulator* (*A-ARR*) genes were up-regulated (Figure 3; Table S2). Additionally, in the SA signal transduction pathway, *TGACG-BINDING FACTOR* (*TGA*) genes had different expression patterns (Figure 3; Table S2). These results indicate that multiple hormones may constitute a complex signal transduction in response to wounding in *A. sinensis*.

### 3.4. Potential Genes Involved in 2-(2-Phenylethyl)chromone Biosynthesis

**Figure 4:**
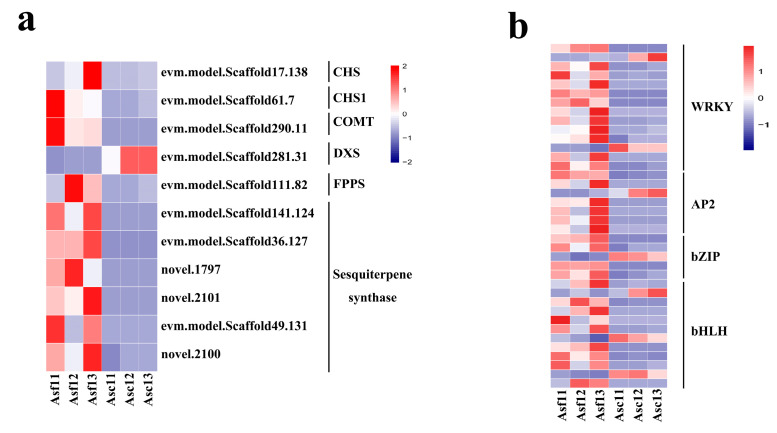
Expression profiling of DEGs and transcription factors. (**a**) Heatmap shows the expression patterns of the DEGs involved in the biosynthesis of 2-(2-phenylethyl)chromones and sesquiterpenes; (**b**) heatmap shows the expression patterns of the DEGs annotated as WRKY, AP2, bZIP, and bHLH TFs. Each row represents each DEG, and each column represents the expression level from different samples. The color from blue to red represents gene expression from low to high.

We found that *chalcone synthase* (*CHS*) and *O*-*methyltransferase* (*OMT*) genes, which are putatively involved in the biosynthesis of 2-(2-phenylethyl)chromones [18], were differentially expressed after mechanical wounding (Figure 4a; Table S3). *CHS* and *CHS1* genes were up-regulated more than 21-fold (Figure 4a; Table S3). Additionally, the *caffeic acid 3-O-methyltransferase* (*COMT*) gene was up-regulated in Asf1 (Figure 4a; Table S3).

### 3.5. DEGs Involved in Sesquiterpene Biosynthesis

In this study, we attempted to discover key genes involved in sesquiterpene biosynthesis. A total of eight DEGs were related to sesquiterpene biosynthesis, which were annotated as the three key enzymes, including 1-deoxy-D-xylulose-5-phosphate synthase (DXS), farnesyl diphosphate (FPP) synthase (FPPS), and sesquiterpene synthase (SS) (Figure 4a). Expression of the *DXS* gene was down-regulated by about 11-fold in Asf1 (Figure 4a; Table S4). DXS catalyzes pyruvate and glyceraldehyde-3-phosphate to synthesize 1-deoxy-D-xylulose-5-phosphate (DXP) (Figure S1), which might be suppressed due to the down-regulation of *DXS*. On the other hand, the *FPPS* gene was up-regulated by about 31-fold (Figure 4a; Table S4). As FPPS converts isopentenyl diphosphate (IPP) to FPP (Figure S1), this up-regulation may contribute to an increase in the accumulation of FPP precursor during the biosynthesis of sesquiterpenes. Moreover, six *SS* genes were up-regulated by more than 11-fold (Figure 4a; Table S4); they convert FPP to sesquiterpenes (Figure S1). Generally, the up-regulation of *FPPS* and *SS* genes may support the biosynthesis of agarwood sesquiterpenes.

### 3.6. Transcription Factors Mediated Regulatory Networks Involved in Sesquiterpene Biosynthesis

**Figure 5:**
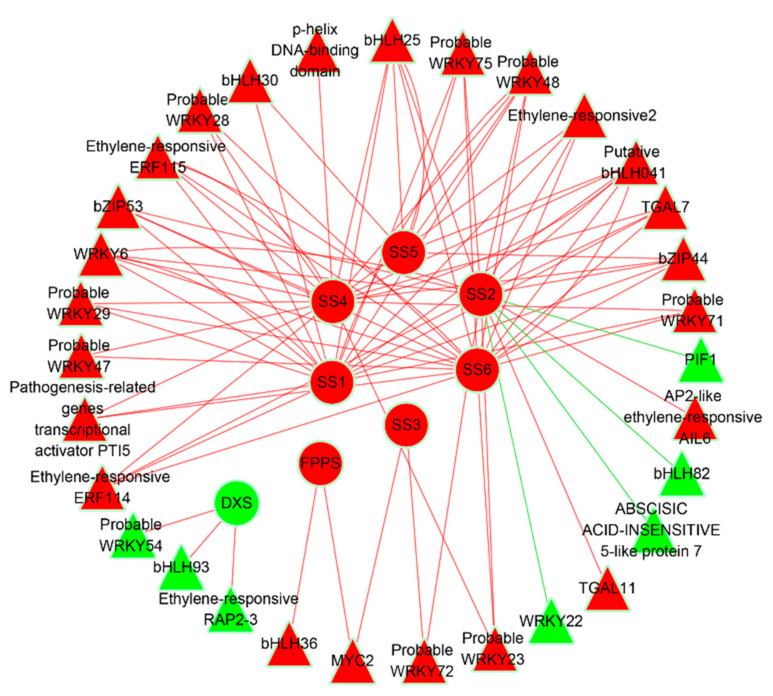
The co-expression network of DEGs involved in sesquiterpene biosynthesis and transcription factors. Red nodes represent up-regulated genes and green nodes represent down-regulated genes. The genes involved in sesquiterpene biosynthesis are represented by circles and transcription factors are represented by triangles. Red lines represent positive correlation and green lines represent negative correlation between genes and transcription factors.

To probe into the regulatory factors of genes involved in sesquiterpene biosynthesis, we constructed a co-expression network with the help of Pearson’s correlation coefficients. In total, 38 DEGs corresponding to TFs, which encoded for WRKY, AP2, bHLH, and bZIP families (Table S5), and 8 DEGs associated with sesquiterpene biosynthesis were co-expressed (Figure 5; Table S6). Most of these TF genes were up-regulated, except the genes that encoded for WRKY54, WRKY22, ethylene-responsive transcription factor RAP2-3, ABSCISIC ACID-INSENSITIVE 5 (ABI5)-like protein 7, bHLH93, bHLH82, and PIF1 (Figure 4b; Table S5). In the network, the expression patterns of genes encoding for WRKY54, ethylene-responsive RAP2-3, and bHLH93 were positively correlated to *DXS* (Figure 5; Table S6), indicating a co-expression. Similarly, *MYC2* and *bHLH36* were positively correlated to *FPPS* (Figure 5; Table S6). On the other hand, the *SS2* gene showed a negative relationship with the expression of genes encoding for WRKY22, ABI5-like protein 7, bHLH82, and PIF1 (Figure 5; Table S6). Interestingly, bHLH TFs could correlate to all DEGs involved in sesquiterpene biosynthesis (Figure 5; Table S6), indicating their critical function in *A. sinensis*. These results indicate that TFs of these four families are likely to play key roles in *A. sinensis* agarwood formation during an early response to mechanical wounding.

### 3.7. RNA-Seq Verification by qRT-PCR

A total of eight genes were randomly selected for qRT-PCR to verify the accuracy and reliability of transcriptome data. The expression of genes indicated the high similarity between the qRT-PCR results and RNA-seq results. The correlation analysis showed a high R^2^ value of 0.7213 between the two techniques (Figure S2; Table S7), and Pearson’s correlation coefficient was 0.85. The results suggest that our RNA-seq data are reliable.

## 4. Discussion

### 4.1. A. sinensis Transcriptome Sequencing

To adapt to mechanical wounding, plants elicit the accumulation of important secondary metabolites by changing cell vitality and by temporary activation of secondary metabolite-related genes [35,36,37]. For example, the mechanical wounding of *Centaurium erythraea* leaves led to an up-regulation of secoiridoid glucoside biosynthetic genes, supporting the accumulation of secoiridoid glucosides [35]. The leaves of *Senna tora* responded to wounding by the induction of genes involved in flavonoid biosynthesis and the accumulation of kaempferol and quercetin [37]. Here, wound-responsive genes were discovered and their expression patterns were identified to explore the regulatory mechanism of agarwood formation in *A. sinensis*. We identified 1596 as differentially expressing (1088 as up- and 508 as down-regulated) between the Asf1 and Asc1 tissues (Figure 1b). Further, GO enrichment analysis showed that these DEGs could participate in various biological functions and activities, including ‘multi-organism process’, ‘cell recognition’, ‘transcription regulator activity’, and ‘oxidoreductase activity’ (Figure 2). Moreover, KEGG enrichment analysis revealed that these DEGs were related to the biosynthesis of secondary metabolites, such as flavonoids, phenylpropanoids, sesquiterpenoids, and triterpenoids (Table 2), which are closely associated with plant responses and tolerance to stresses [38,39,40,41,42]. Hence, up-regulation of these secondary metabolite-related genes might imply that mechanical wounding could induce the biosynthesis of secondary metabolites in *A. sinensis* (Table 2). Further, the universal down-regulation of photosynthesis-related genes is an adaptive response to environmental stresses [43,44,45]. In our study, genes involved in the photosynthesis pathway were down-regulated (Table 2), indicating that they may play important roles in *A. sinensis* wound response. Overall, DEGs offer a large amount of genetic information for characterizing the key genes associated with *A. sinensis* agarwood formation during the response to mechanical wounding.

### 4.2. Jasmonic Acid and Salicylic Acid Have Potential Regulatory Roles in Agarwood Formation

Phytohormones have pivotal roles in plant growth and development, response to environmental stresses, and secondary metabolite biosynthesis [46,47,48,49]. Further, JA signaling plays a pivotal role in agarwood formation [23,50,51]. Our data indicated that the *COI1* gene was down-regulated, while the *MYC2* gene was up-regulated in Asf1 (Figure 3; Table S2). In the JA signal transduction, COI1 acts as both a JA receptor and the F-box component of the SCF^COI1^ complex [52]. Jasmonate ZIM-domain (JAZ) proteins directly target SCF^COI1^ E3 ubiquitin ligase, linking ubiquitin-mediated protein degradation, which liberates MYC2 to allow the induction of JA-responsive genes [52]. Further, *AsCOI1* likely plays a role in agarwood formation [53], and AsMYC2 could also be involved in the biosynthesis of agarwood sesquiterpenes by regulating sesquiterpene synthases [23]. Consistent with these studies, our data indicate that the JA signaling may have functions in the process of agarwood formation in *A. sinensis* during an early response to mechanical wounding. Further, the JA signal pathway might interact with other signal pathways, including SA, to optimize plant defense response against stresses [54,55,56]. Moreover, SA plays an important role in the process of agarwood formation during stresses [10,50,57]. In this study, genes encoding for TGA were differently expressed between Asf1 and Asc1 (Figure 3; Table S2), indicating that SA may participate in agarwood formation in the early response to mechanical wounding. Overall, our results indicate that JA and SA may have potential regulatory roles in wound-induced agarwood formation.

### 4.3. Key Genes Associated with 2-(2-Phenylethyl)chromone Biosynthesis in A. sinensis

The main ingredients of agarwood are 2-(2-phenylethyl)chromones [4]. Hence, studies have paid much attention to the molecular mechanism of their biosynthesis. Chalcone synthase (CHS, a type III polyketide synthase) and O-methyltransferase (OMT) play critical roles in their biosynthesis [18,58]. The 2-(2-phenylethyl)chromones are composed of flindersia-type 2-(2-phenylethyl)chromones (FTPECs), and OMTs catalyze the step that leads 2-(2-phenylethyl)chromone scaffold to form structurally diverse FTPECs [59]. In the present study, *CHS* and *COMT* were significantly up-regulated during the response to mechanical wounding (Figure 4a; Table S3), implying that these genes might participate in the 2-(2-phenylethyl)chromone synthesis in wounded *A. sinensis*. These findings are consistent with those of Wang et al. [18], who investigated the influence of salt stress on *A. sinensis* calli, and demonstrated that *CHS* and *OMTs* may support the biosynthesis of 2-(2-phenylethyl)chromones. Nevertheless, Wang et al. [60] asserted that a diarylpentanoid-producing polyketide synthase (PECPS) could play a crucial role in the biosynthesis of C6-C5-C6 scaffold of diarylpentanoid, the common precursor of 2-(2-phenylethyl)chromones. Clearly, the biosynthesis pathway of 2-(2-phenylethyl)chromones warrants further investigation.

### 4.4. Key Genes Associated with Sesquiterpene Biosynthesis in A. sinensis

Sesquiterpenes are the important components of agarwood, and their content could be used to judge the agarwood quality [4,5]. Terpenes are biosynthesized in plants from the two pathways, the mevalonic acid pathway (MVA pathway) in the cytoplasm, and methylerythritol 4-phosphate pathways (MEP pathway) in the plastids [61]. Correspondingly, the former contributes to the biosynthesis of sesquiterpenoids, triterpenoids, and sterols, while the latter contributes to the biosynthesis of diterpenoids, monoterpenoids, and carotenoids [62]. Also, a cross-talk between these two different pathways through the common C5 isoprene unit, IPP, and dimethylallyl diphosphate (DMAPP), has been reported [63,64,65].

Sesquiterpene synthesis is positively associated with the expression of synthases, including DXS, HMGR, 3-hydroxy-3-methylglutaryl CoA synthase (HMGS), phosphomevalonate kinase (PMK), FPPS, and SS [19,66,67]. In this study, the *DXS* gene was down-regulated (Figure 4a; Table S4). However, *FPPS* and *SS* genes were up-regulated (Figure 4a; Table S4), indicating the sesquiterpene biosynthetic process may be enhanced to support the biosynthesis and accumulation of sesquiterpenes. Our data offer critical clues for future studies on the sesquiterpene biosynthesis pathway in *A. sinensis* during the early response to mechanical wounding.

### 4.5. A Transcriptomic Network Underlying the Regulation of Sesquiterpene Biosynthesis

Recently, many reports demonstrated the role of TFs in stress responses, for example by regulating secondary metabolites [68]. Previous reports demonstrated that TF families, including AP2, WRKY, bZIP, and bHLH, could function in sesquiterpene biosynthesis and affect the sesquiterpene content [23,51,69,70]. For example, the expression of most WRKY TFs reached the maximum value in the agarwood, with a positive correlation to sesquiterpenoid biosynthetic genes [8]. GaWRKY1 functioned in cotton sesquiterpene biosynthesis by interacting with the promoter of *CAD1*-*A*, a member of the (+)-δ-Cadinene synthase (CAD1) gene family [71]. AsWRKY44 could directly bind the *agarwood sesquiterpene synthase 1* (*ASS1*) promoter and repress its activity to negatively regulate sesquiterpene biosynthesis in agarwood [51]. Based on the differential expression of WRKY TFs (Figure 4b; Table S5), we infer that they may have potential functions in agarwood formation and also participate in the early response to mechanical wounding. Additionally, MYC2, a bHLH TF family member, could directly bind to *TPS21* and *TPS11* promoters to activate their expression, and, thus, promote sesquiterpene production [72]. In our study, the majority of the bHLH TFs showed an up-regulation while genes that encoded for bHLH93, bHLH82, and PIF1 were down-regulated (Figure 4b; Table S5). These results indicated that bHLH TFs may regulate agarwood formation in the early response to wounding. Also, AP2 and bZIP TFs were suggested to regulate terpene biosynthesis-related genes during volatile terpenoid formation in other plants [69,73,74,75,76,77]. In *Catharanthus roseus*, ORCA3 induced the expression of DXS to contribute to the biosynthesis of terpenoid indole alkaloids [73]. Further, in *Bupleurum chinense*, BcbZIP134 could negatively regulate saikosaponin biosynthesis [76]. In our investigation, the majority of bZIP and AP2 TFs were up-regulated, whereas genes that encoded for ethylene-responsive RAP2-3 and ABI5-like protein 7 were down-regulated (Figure 4b; Table S5) during wound-induced agarwood formation. However, information on the relationship between TFs and genes of sesquiterpene biosynthesis in *A. sinensis* has been limited. Based on a co-expression network, our data found that WRKY, AP2, bHLH, and bZIP TFs could be potentially involved in sesquiterpene biosynthesis by activating or repressing key genes, especially bHLH TFs (Figure 5; Table S6). These results provide new clues for further studies of the TFs involved in sesquiterpene biosynthesis in *A. sinensis*.

## 5. Conclusions

RNA-seq analysis of early response to wounding of specific xylem tissues (Asf1 and Asc1) of *A. sinensis* provides molecular insight into wound-induced agarwood formation. A total of 1596 DEGs were identified, which included genes related to secondary metabolism, such as flavonoids, phenylpropanoids, sesquiterpenoids, and triterpenoids. The network of sesquiterpene biosynthesis genes and TF genes was constructed, which showed that bHLH TFs, during wounding response, co-expressed with all the genes involved in sesquiterpene biosynthesis, including *DXS*, *FPPS*, and *SS* genes; this indicates a critical role of the bHLH family in sesquiterpene biosynthesis. Taken together, these findings enhance our understanding of the regulation of sesquiterpene biosynthesis. It also provides a comprehensive transcriptomic dataset that would be valuable for subsequent gene discoveries, understanding transcriptional regulation, and for genomics research about wound-induced agarwood formation in *Aquilaria* trees.

## Figures and Tables

**Table 1:** Summary of RAN-seq data of *A. sinensis* after mechanical wounding.

Sample	Raw Reads	Clean Reads	Mapped to Genome	Q20 (%)	Q30 (%)	GC (%)
Asc11	48,158,840	45,845,016	41,524,528 (90.58%)	98.33	94.85	46.96
Asc12	49,229,480	48,487,386	44,432,205 (91.64%)	97.59	93.02	46.49
Asc13	55,055,938	52,975,166	48,697,477 (91.93%)	98.20	94.51	46.32
Asf11	46,747,414	43,317,726	38,986,805 (90.00%)	98.34	94.86	46.17
Asf12	50,747,240	47,754,238	43,633,605 (91.37%)	98.10	94.27	46.50
Asf13	45,935,934	44,470,980	36,366,124(81.77%)	97.70	93.60	47.84

**Table 2:** Significantly enriched KEGG pathways of DEGs.

KEGG_ID	Pathway Name	Number	Up	Down
pop00941	Flavonoid biosynthesis	14	14	0
pop00940	Phenylpropanoid biosynthesis	24	24	0
pop00480	Glutathione metabolism	12	12	0
pop00909	Sesquiterpenoid and triterpenoid biosynthesis	6	6	0
pop00400	Phenylalanine, tyrosine and tryptophan biosynthesis	8	8	0
pop00360	Phenylalanine metabolism	7	6	1
pop00195	Photosynthesis	9	0	9

## Data Availability

The transcriptome data for *A. sinensis* reported in this paper have been deposited at the Genome Sequence Archive in BIG Data Center (BIG Data Center Members, 2019), Beijing Institute of Genomics (BIG), Chinese Academy of Sciences, under accession numbers CRA008994, and are publicly available at https://bigd.big.ac.cn/gsa (accessed on 21 November 2022).

## Supplementary Materials

The following supporting information can be downloaded at: https://www.mdpi.com/article/10.3390/genes14020464/s1, Figure S1: Schematic diagram of sesquiterpenoid biosynthesis pathway activated in *A. sinensis* responding to mechanical wounding; Figure S2: The comparison of the expression levels of eight genes between RNA-seq and qRT-PCR; Table S1: Primer pairs of selected genes for qRT-PCR validation; Table S2: DEGs involved in hormone signal transduction; Table S3: DEGs involved in 2-(2-phenylethyl)chromone biosynthesis; Table S4: DEGs involved in sesquiterpene biosynthesis; Table S5: DEGs related to transcription factors; Table S6: Expression profiles of 38 TFs were correlated with genes involved in sesquiterpene biosynthesis; Table S7: qRT-PCR validation of selected genes obtained by RNA-seq in *A. sinensis* subjected to mechanical wounding.
