# Supplementary material for: RNA-Sequencing Reveals the Involvement of Sesquiterpene Biosynthesis Genes and Transcription Factors during an Early Response to Mechanical Wounding of Aquilaria sinensis
Source: Genes (Basel). 2023 Feb 11;14(2):464. doi: 10.3390/genes14020464 (PMC9957285; doi:10.3390/genes14020464)
Supplement: Supplementary file 1 [file genes-14-00464-s001.zip › genes-2167277-Supplementary.pdf]

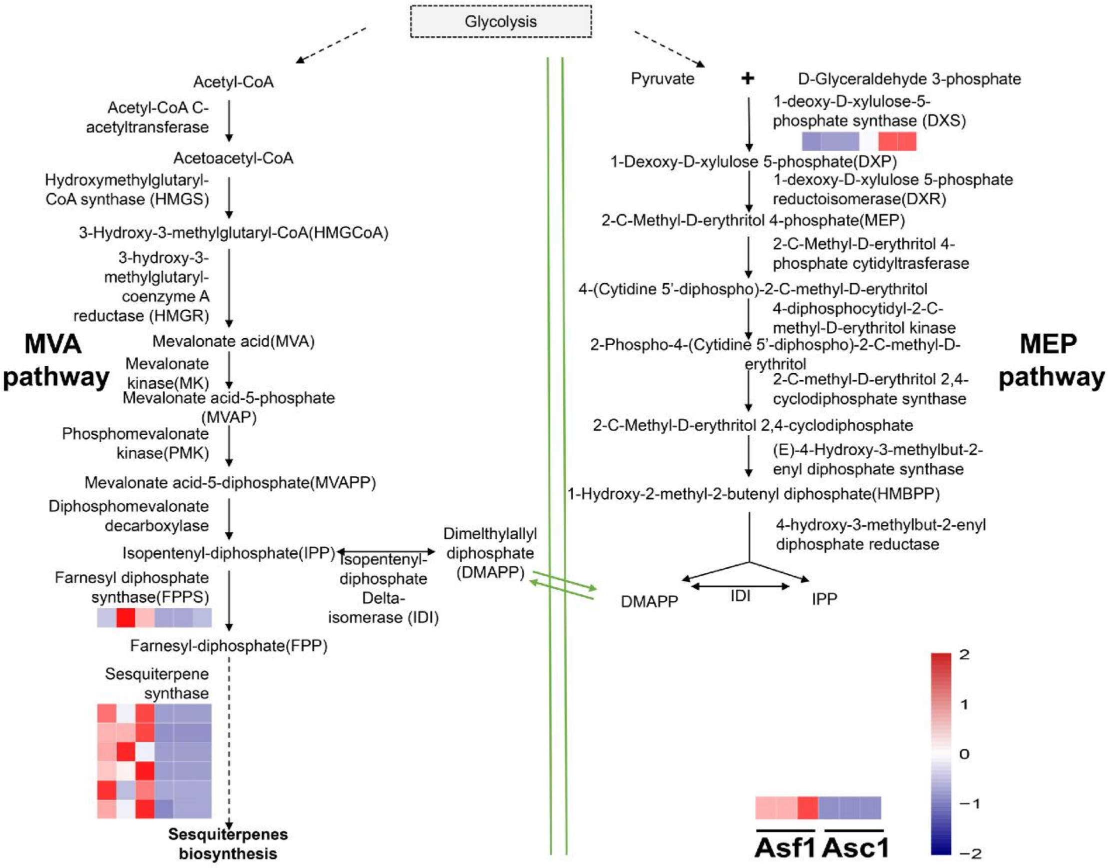

Figure S1. Schematic diagram of sesquiterpenoid biosynthesis pathway activated in *A. sinensis* responding to mechanical wounding. Each row represents each DEG, and each column represents the expression level from different samples. The color from blue to red represents gene expression from low to high

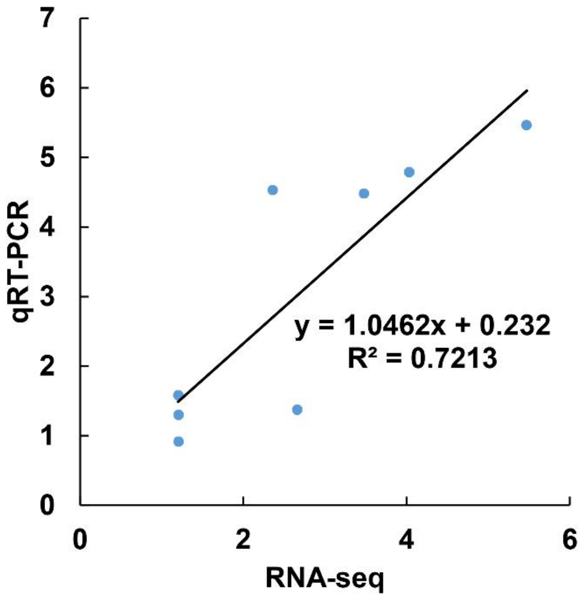

Figure S2. The comparison of the expression levels of eight genes between RNA-seq and qRT-PCR. The x-axis and y-axis represent the log_2(fold change) values by RNA-seq and qRT-PCR for each gene, respectively

Table S1. Primer pairs of selected genes for qRT-PCR validation

Gene ID	Primer pairs
Ubiquitin	F:CCAGATCAATCTTGATTCCACC
	R:ACCTAAACCAGAGCCCCCTA
MYB2	F:GCAACCACCAATAGCCCTGT
	R:TCACCACTCCAATTCCAAACA
BHLH25	F:TCCTTGTGATTCCTTCATTTCG
	R:TGAGCCTCTCTCGTCTCTTCCT
WRKY75	F:TGATGGCTATCGTTGGAGGA
	R:CATTTGGCTGAGGATGTGCT
SSL6	F:ATCCCAAGACCAAGAGCACC
	R:GAATATTATCCGGCAAGCCAG
SYP121	F:AGCGAGATTCAGGAGAGGCA
	R:CGACCAAGATGATGATGAGGAG
PHT1-4	F:AGAGGCGGAGGAAGAGAAAGT
	R:TGAGGAGAGCGATGAGGGTT
CYP749A20	F:TTATATCCCCCTGTTTTTGTCG
	R:TGTTCTTCGTTGCTTTCGCT
SRG1	F:AGCCGGTCATTAGGGTCCAA
	R:CATCATCTCCTCCCGAAGTTTT
C4	F:GAGTCCAATACGAGCCCCAA
	R:CGTCGCTTCAATTTCCCACC
LHT1	F:TACATTCCCTTTCTTCGGTGG
	R:GCTTGGATGATGATTTGCCTC

Table S2. DEGs involved in hormone signal transduction

Gene ID	Gene description	Fold change
evm.model.Scaffold43.250	AUX/IAA	0.33
evm.model.Scaffold4.216	AUX/IAA	0.07
evm.model.Scaffold12.178	SAUR	0.08
evm.model.Scaffold13.104	SAUR	563.62
evm.model.Scaffold2.51	SAUR	0.06
evm.model.Scaffold74.8	SAUR	0.07
evm.model.Scaffold3.405	SAUR	0.01
evm.model.Scaffold8.391	SAUR	128.06
evm.model.Scaffold11.46	SAUR	0.05
evm.model.Scaffold16.5	COI1	0.38
evm.model.Scaffold206.78	MYC2	44.77
evm.model.Scaffold251.7	A-ARR	8.22
evm.model.Scaffold3.493	A-ARR	12.13
evm.model.Scaffold548.8	PYR/RYL	0.31
evm.model.Scaffold50.26	EIN3	0.48
evm.model.Scaffold6.539	BAK1	4.24
evm.model.Scaffold2.236	BKI1	19.76
evm.model.Scaffold64.58	TGA	45.75
evm.model.Scaffold192.66	TGA	557.39
evm.model.Scaffold31.203	TGA	0.21

Table S3. DEGs involved in 2-(2-phenylethyl)chromone biosynthesis

Gene ID	Gene description	Fold change
evm.model.Scaffold17.138	CHS	69.57
evm.model.Scaffold61.7	CHS1	21.66
evm.model.Scaffold290.11	COMT	1472.86

Table S4. DEGs involved in sesquiterpene biosynthesis

Gene ID	Gene description	Fold change
evm.model.Scaffold281.31	DXS, 1-deoxy-D-xylulose-5-phosphate synthase	0.09
evm.model.Scaffold111.82	FPPS, farnesyl diphospate synthase	31.03
evm.model.Scaffold141.124	SS1, sequiterpene synthase1	27819.51
evm.model.Scaffold36.127	SS2, sequiterpene synthase2	1242.42
novel.1797	SS3, sequiterpene synthase3	538.21
novel.2101	SS4, sequiterpene synthase4	127.89
evm.model.Scaffold49.131	SS5, sequiterpene synthase5	194.56
novel.2100	SS6, sequiterpene synthase6	11.77

Table S5. DEGs related to transcription factors

Gene ID	Gene description	TF family	Fold change
evm.model.Scaffold10.36	Probable WRKY72	WRKY	208.77
evm.model.Scaffold113.34	Probable WRKY54	WRKY	0.06
evm.model.Scaffold131.93	Probable WRKY48	WRKY	16.63
evm.model.Scaffold135.59	Probable WRKY48	WRKY	40.02
evm.model.Scaffold171.22	Probable WRKY28	WRKY	196.91
evm.model.Scaffold19.164	WRKY6	WRKY	106.24
evm.model.Scaffold271.5	Probable WRKY72	WRKY	1120.23
evm.model.Scaffold3.221	Probable WRKY29	WRKY	297.41
evm.model.Scaffold49.21	WRKY6	WRKY	158.01
evm.model.Scaffold7.243	Probable WRKY47	WRKY	16.95
evm.model.Scaffold7.283	Probable WRKY23	WRKY	3.64
evm.model.Scaffold7.87	WRKY22	WRKY	0.17
evm.model.Scaffold8.405	Probable WRKY71	WRKY	989.44
evm.model.Scaffold9.167	Probable WRKY75	WRKY	43.26
evm.model.Scaffold112.45	AP2-like ethylene-responsive AIL6	AP2	9.54
evm.model.Scaffold13.71	Ethylene-responsive ERF115	AP2	221.10
evm.model.Scaffold131.31	Ethylene-responsive RAP2-3	AP2	0.26
evm.model.Scaffold307.28	Ethylene-responsive ERF114	AP2	167.45
evm.model.Scaffold38.135	Pathogenesis-related genes transcriptional activator PTI5	AP2	240.75
evm.model.Scaffold53.50	Ethylene-responsive2	AP2	86.51
evm.model.Scaffold74.54	Ethylene-responsive ERF115	AP2	13551.73
evm.model.Scaffold192.66	TGAL7	bZIP	557.39
evm.model.Scaffold2.100	bZIP44	bZIP	4.47
evm.model.Scaffold6.343	ABSCISIC ACID-INSENSITIVE 5-like protein 7	bZIP	0.44
evm.model.Scaffold64.58	TGAL11	bZIP	45.75
evm.model.Scaffold7.303	bZIP53	bZIP	2.36
evm.model.Scaffold1.376	bHLH25	bHLH	15.66
evm.model.Scaffold2.401	bHLH93	bHLH	0.14
evm.model.Scaffold206.78	MYC2	bHLH	44.77
evm.model.Scaffold22.131	p-helix DNA-binding domain	bHLH	202.84
evm.model.Scaffold28.184	bHLH25	bHLH	27.85
evm.model.Scaffold29.150	Putative bHLH041	bHLH	57.45
evm.model.Scaffold46.105	bHLH82	bHLH	0.26
evm.model.Scaffold5.546	Putative bHLH041	bHLH	169.70
evm.model.Scaffold6.47	bHLH25	bHLH	44.40
evm.model.Scaffold60.31	bHLH30	bHLH	18.70
evm.model.Scaffold8.93	PIF1	bHLH	0.40
evm.model.Scaffold95.41	bHLH36	bHLH	123.19

Table S6. Expression profiles of 38 TFs were correlated with genes involved in sesquiterpene biosynthesis.

Gene ID	Gene description	TF family	Pearson correlation coefficient (cor)							
			DXS	FPPS	SS1	SS2	SS3	SS4	SS5	SS6
evm.model.Scaffold10.36	Probable WRKY72	WRKY	-	-	-	0.963690981	-	-	-	-
evm.model.Scaffold113.34	Probable WRKY54	WRKY	0.907162824	-	-	-	-	-	-	-
evm.model.Scaffold131.93	Probable WRKY48	WRKY	-	-	0.972177523	0.96489446	-	0.990938546	-	0.99541661
evm.model.Scaffold135.59	Probable WRKY48	WRKY	-	-	0.919425369	-	-	-	0.985888134	-
evm.model.Scaffold171.22	Probable WRKY28	WRKY	-	-	0.939651576	-	-	0.980523982	-	0.986222882
evm.model.Scaffold19.164	WRKY6	WRKY	-	-	0.913988827	0.946653757	-	-	-	-
evm.model.Scaffold271.5	Probable WRKY72	WRKY	-	-	-	-	0.95225151	-	-	-
evm.model.Scaffold3.221	Probable WRKY29	WRKY	-	-	0.901746174	-	-	0.967652899	-	0.966884949
evm.model.Scaffold49.21	WRKY6	WRKY	-	-	0.964555044	0.907959875	-	0.977026404	-	0.992896913
evm.model.Scaffold7.243	Probable WRKY47	WRKY	-	-	-	-	-	0.953304528	-	0.90968441
evm.model.Scaffold7.283	Probable WRKY23	WRKY	-	-	-	0.922849089	-	0.96982079	-	0.935549574
evm.model.Scaffold7.87	WRKY22	WRKY	-	-	-	-0.91396498	-	-	-	-
evm.model.Scaffold8.405	Probable WRKY71	WRKY	-	-	0.96553656	-	-	0.954592648	-	0.983509094
evm.model.Scaffold9.167	Probable WRKY75	WRKY	-	-	0.982333853	0.917061171	-	-	0.939147262	0.924077332
evm.model.Scaffold112.45	AP2-like ethylene-responsive AIL6	AP2	-	-	-	0.912427968	-	-	-	-
evm.model.Scaffold13.71	Ethylene-responsive ERF115	AP2	-	-	0.91415969	-	-	0.973636944	-	0.974112578
evm.model.Scaffold131.31	Ethylene-responsive RAP2-3	AP2	0.988060865	-	-	-	-	-	-	-
evm.model.Scaffold307.28	Ethylene-responsive ERF114	AP2	-	-	0.904946609	0.951301863	-	0.996189451	-	0.968301068
evm.model.Scaffold38.135	Pathogenesis-related genes transcriptional activator PTI5	AP2	-	-	0.934418781	-	-	0.957840544	-	0.975572503
evm.model.Scaffold53.50	Ethylene-responsive2	AP2	-	-	0.959079187	0.929550036	-	0.989814037	-	0.994833018
evm.model.Scaffold74.54	Ethylene-responsive ERF115	AP2	-	-	-	-	-	0.948120286	-	0.943052971
evm.model.Scaffold192.66	TGAL7	bZIP	-	-	0.913527945	0.999158294	-	0.955937791	-	0.928621714
evm.model.Scaffold2.100	bZIP44	bZIP	-	-	0.992545052	0.91620193	-	0.933670535	0.931199821	0.978479476
evm.model.Scaffold6.343	ABSCISIC ACID-INSENSITIVE 5-like protein 7	bZIP	-	-	-	-0.92299566	-	-	-	-
evm.model.Scaffold64.58	TGAL11	bZIP	-	-	-	0.9675408	-	-	-	-
evm.model.Scaffold7.303	bZIP53	bZIP	-	-	0.975013119	0.981307067	-	0.973996225	-	0.98105101
evm.model.Scaffold1.376	bHLH25	bHLH	-	-	-	-	-	0.907613919	-	-
evm.model.Scaffold2.401	bHLH93	bHLH	0.950768076	-	-	-	-	-	-	-

evm.model.Scaffold206.78	MYC2	bHLH	-	0.921214566	-	-	0.917845566	-	-	-
evm.model.Scaffold22.131	p-helix DNA-binding domain	bHLH	-	-	-	-	-	0.903808426	-	-
evm.model.Scaffold28.184	bHLH25	bHLH	-	-	-	-	-	-	0.951351038	-
evm.model.Scaffold29.150	Putative bHLH041	bHLH	-	-	0.985054475	-	-	0.942545604	0.927974945	0.98158514
evm.model.Scaffold46.105	bHLH82	bHLH	-	-	-	-0.94977593	-	-	-	-
evm.model.Scaffold5.546	Putative bHLH041	bHLH	-	-	-	0.98747536	-	0.97209321	-	0.931595667
evm.model.Scaffold6.47	bHLH25	bHLH	-	-	0.977789135	0.928205057	-	-	0.923795183	0.918901095
evm.model.Scaffold60.31	bHLH30	bHLH	-	-	0.961825798	-	-	-	0.993148317	-
evm.model.Scaffold8.93	PIF1	bHLH	-	-	-	-0.93270524	-	-	-	-
evm.model.Scaffold95.41	bHLH36	bHLH	-	0.941851497	-	-	-	-	-	-

Table S7. qRT-PCR validation of selected genes obtained by RNA-seq in *A. sinensis* subjected to mechanical wounding.

Gene ID	Gene name	Gene_description	log_2(fold change)	
			RNA-seq	qRT-PCR
evm.model.Scaffold306.41	MYB2	Transcription factor MYB108	2.66	1.38
evm.model.Scaffold6.47	BHLH25	Transcription factor bHLH25	5.47	5.46
evm.model.Scaffold38.68	SSL6	Protein STRICTOSIDINE SYNTHASE-LIKE 6	2.36	4.53
evm.model.Scaffold33.243	SYP121	Syntaxin-121	1.20	1.58
evm.model.Scaffold22.170	PHT1-4	Probable inorganic phosphate transporter 1-5	3.48	4.49
evm.model.Scaffold31.97	CYP749A20	Cytochrome P450 CYP749A22	1.20	0.92
evm.model.Scaffold11.100	C4	Delta-guaiene synthase 3	1.20	1.30
evm.model.Scaffold10.221	LHT1	Lysine histidine transporter 1	4.03	4.79
